# Tuberculosis laboratory capacity building in the WHO African Region: The past, the present and the future: A Viewpoint

**DOI:** 10.1371/journal.pgph.0004979

**Published:** 2025-11-11

**Authors:** Jean de Dieu Iragena, Achilles Katamba, Dissou Affolabi, Moses Joloba, Willy Ssengooba

**Affiliations:** 1 Department of Immunology and Molecular Biology, Makerere University College of Health Sciences, School of Biomedical Sciences Kampala, Kampala, Uganda; 2 Department of Medicine, Makerere University School of Medicine, Clinical Epidemiology and Biostatistics Unit, Uganda Implementation Research Consortium, Kampala, Uganda; 3 National Teaching Hospital for Tuberculosis and Respiratory Diseases, Cotonou, Republic of Benin; 4 National Tuberculosis Program, Cotonou, Republic of Benin; 5 Makerere University Lung Institute, Kampala, Uganda; 6 Makerere University, Biomedical Research Center, Kampala, Uganda; 7 Department of Medical Microbiology, Makerere University College of Health Sciences Kampala, Kampala, Uganda; Human Sciences Research Council, SOUTH AFRICA

## Abstract

Tuberculosis remains a leading infectious disease killer in the World Health Organization African Region, with 2.5 million cases and 404,000 deaths in 2023, including 112,000 people with HIV. There is slow progress with only 42% of the 75% targeted reduction in death by 2025. Out of 60,266 estimated multidrug-resistant TB cases in 2023, only 22,515 were notified. Laboratory diagnostic services in the African region still need urgent attention. By 2005, the new smear-positive case detection rate was nearly 51%, falling short of the 70% target. By 2015, the benchmark for one microscopy center per 100,000 population was reached in some Member States, but gaps remained in culture and drug susceptibility testing coverage. Molecular tests were adopted, however there is slow uptake among countries to use them as initial diagnostic tests. The Global Laboratory Initiatives were established in 2007 and 2013 globally and in the WHO African Region respectively to enhance access to quality-assured TB laboratory services. The WHO TB Supranational Reference Laboratory (SRL) Network was established in 1994 and expanded to the African region, including South Africa, Uganda, and Benin. The nomination of Mozambique and Rwanda in 2021 as candidate SRLs aims to strengthen this network. Future perspectives involve leveraging the established TB laboratory networks to integrate systems for diagnosing multiple diseases while enhancing efficiency. Advocacy for increased funding is vital for sustaining gains in the laboratory capacities, advancing universal health coverage and enhancing health outcomes in the African region. Here we discuss the TB laboratory capacity building in the WHO African region, focusing on the past, present and the future perspectives. We suggest recommendation towards sustaining and strengthening the existing achievements, while accelerating the laboratory interventions towards the End TB Strategy.

## Introduction

In 2023, almost 1.9 million people (out of an estimated 2.5 million people who fell ill with TB) with new and relapsed TB cases were diagnosed and notified in the World Health Organization African Region (WHO/AFR), representing 76% of notified TB cases and leaving a gap of 24% missed TB cases [1]. Although there has been a 24% decline in TB incidence since 2015, this reduction is not sufficient to meet the 50% target by 2025 [1].

Of the reported cases in the WHO/AFR, 54% of individuals newly diagnosed with TB were tested using the WHO-recommended rapid diagnostics (WRDs), which falls far short of the United Nations (UN) high-level meeting targets of 100% of TB diagnoses being initially tested with a WRD by 2027 [2]. Among pulmonary TB cases, 69% were bacteriologically confirmed. Of these, 70% of new cases and 81% of previously treated cases were tested for rifampicin susceptibility. Additionally, 61% of individuals with rifampicin-resistant, bacteriologically-confirmed pulmonary TB were tested for susceptibility to fluoroquinolones, leaving a gap of 39% of rifampicin/multi-drug resistant TB (RR/MDR-TB) cases not tested for second-line DST, making it difficult to diagnose TB and all forms of TB resistance [1].

Tuberculosis (TB) remains the world’s leading infectious disease killer, with 404,000 TB deaths occurring in 2023 (Over 30% of global TB deaths) in the WHO/AFR [1]. Despite a 42% reduction in TB deaths since 2015, this progress is still slow to achieve the 75% target by 2025.

In 2023, the WHO/AFR reported 25.6 million PLHIV [3]. Of this, 1.8%, approximately 461,000 individuals, were newly diagnosed with TB and 314,000 (68%) of them were notified [1].

Tuberculosis continues to be a significant cause of death among people living with HIV (PLHIV), highlighting the persistent challenge of HIV-TB co-infection in Africa [4].

Drug-resistant TB (DR-TB) remains a public health crisis. In 2023, with 60,266 MDR/RR-TB estimated cases in the WHO/AFR, 22,515 DR-TB cases were diagnosed, leaving 37,751 (63%) cases undiagnosed or unreported. A key contributing factor is limited access to rapid diagnostics, which currently stands at only 54% new and relapse tested with rapid diagnostics at initial diagnosis in the WHO/AFR. Strengthened surveillance, diagnostics, and case-finding in the WHO/AFR are urgently needed to close this gap.

To address these challenges, there is a call to accelerate efforts to meet the End TB Strategy targets by enhancing diagnostic access and scaling up of rapid molecular diagnostics to ensure early detection and appropriate treatment. Over the past three decades, the implementation of laboratory diagnostics has significantly contributed to better control of the TB epidemic worldwide, particularly in the WHO/AFR. Since the WHO declared TB a global emergency in 1993 and launched the five-element Directly Observed Treatment Short-course **(**DOTS) strategy in 1994–1995, with its adoption in 2000 [5], there has been progress in enhancing laboratory capacity. This advancement has been propelled by the evolution of various WHO strategies following DOTS, such as the Stop TB Strategy [6] and the End TB Strategy [7]. However, much work remains to sustain the achievements made so far and to move towards ending TB by 2035 and beyond.

This viewpoint paper aims to highlight the efforts made to strengthen TB laboratories in the WHO/AFR. First, it reviews the evolution of TB laboratory diagnostics over three decades, starting with the DOTS Strategy (1994–2005), followed by the Stop TB Strategy (2006–2015), and the End TB Strategy (2016–2035). Second, it highlights global and regional initiatives including the SRL Network aimed at advancing TB diagnostics in the WHO/AFR. Finally, it discusses future perspectives for laboratory integration to diagnose multiple diseases beyond TB, building on the existing infrastructure.

The DOTS and Stop TB Strategy eras are considered the past, while the End TB Strategy era represents the present. The future perspective encompasses the period following 2035.

### The World Health Organization Strategies for Tuberculosis

To facilitate understanding of the evolution of tuberculosis (TB) control efforts, the following table presents a comparative overview of the major WHO strategies—DOTS, Stop TB, and End TB (**Table 1**). These strategies represent distinct phases in global TB response, each with specific objectives, challenges, achievements, and areas requiring further improvement. The DOTS Strategy laid the foundation for standardized TB care, the Stop TB Strategy expanded the scope to include drug-resistant TB and HIV co-infection, and the End TB Strategy aims to eliminate TB as a public health threat by 2035 through ambitious targets for incidence, mortality, and financial protection. This summary provides a concise reference to appreciate the progress made and the gaps that remain.

**Table 1:** Summary of WHO TB Strategies [5–7].

Strategy	Objectives	Challenges	Achievements	Areas Needing Improvement
DOTS (1994–2005)	Detect ≥70% of smear-positive TB cases Achieve ≥85% cure rate	Lack of national laboratory standards Limited culture/DST capacity HIV impact on TB incidence Inadequate funding and staffing	Improved microscopy coverage in some HBCs TB declared emergency in WHO/AFR in 2005	Culture and DST access Drug resistance surveillance National policies for laboratory expansion
Stop TB (2006–2015)	Reduce TB incidence and halve prevalence/mortality by 2015 Align with MDGs	Poor laboratory quality in some countries Inadequate infrastructure for culture/DST Weak EQA systems	59.5% countries met microscopy benchmark Introduction of LPA and GeneXpert Expansion of laboratory networks	Culture and DST coverage still low Integration of new diagnostics outside NTPs
End TB (2016–2035)	Reduce TB incidence by 80% compared to 2015 Reduce TB mortality by 90% compared to 2015 Eliminate catastrophic costs for TB-affected families	Gaps in WRD coverage Limited second-line DST Notification gaps	Framework of indicators for laboratory strengthening Expansion of SRL network GLI Africa regional support	Full WRD coverage Second-line DST access Integration of diagnostics for multiple diseases

Key: DOTS, Directly Observed Treatment, Short-course; DST, Drug Susceptibility Testing; EQA, External Quality Assessment; GLI, Global Laboratory Initiative; LPA, Line Probe Assay; MDGs, Millennium Development Goals; NTPs, National Tuberculosis Programs; SRL, Supranational Reference Laboratory; and WRD, WHO-recommended Rapid Diagnostic.

## The Past

### Directly Observed Treatment Short course (DOTS), (1994–2005)

During the period of 1994–2005, the performance of laboratory diagnostic services urgently needed improvement in most High TB Burden Countries (HBCs), including those in the WHO/AFR [8]. At that time, national standards for laboratory procedures were absent in many countries.

The main objective was to identify at least 70% of individuals with sputum smear-positive TB and to ensure a cure rate of at least 85%.

#### Smear microscopy.

To achieve this, countries in the WHO African Region focused on expanding access to microscopy services, decentralizing smear microscopy to peripheral health facilities, and training laboratory personnel in sputum smear techniques. External Quality Assessment (EQA) programs were introduced to ensure diagnostic accuracy, and national TB programs prioritized the establishment of one microscopy center per 100,000 population [9]. These interventions were specifically designed to improve case detection through smear microscopy.

By the close of 2005, the new smear-positive case detection rate in the WHO/AFR was nearly 51%, falling short of the 70% target [10]. Despite improvements in the geographical coverage of laboratory services in 22 HBCs, significant gaps remained. Only nine HBCs reported external quality assessment (EQA) coverage exceeding 50% of designated reference laboratories [11].

#### Culture and DST.

Laboratories were anticipated to be well-equipped and adequately staffed to detect patients with TB and drug-resistant TB. However, building capacity to culture *Mycobacterium tuberculosis* and perform drug susceptibility testing (DST) presented substantial challenges for many national TB programs (NTPs). Investments in laboratory infrastructure and biosafety were critically needed.

In the WHO/AFR, South Africa was exceptional in reporting good coverage of culture facilities, exceeding the minimum of one culture facility per five million population [11,12].

However, over half of the population in the WHO/AFR had limited access to culture services. Most countries lacked national policies to expand culture and DST services and the technical capacity to implement and support such services [13]. Major barriers included a lack of staff, transportation issues, and inadequate funding [14].

In the context of these few achievements, TB control in the WHO/AFR faced other severe challenges, the greatest of which was the impact of HIV on increasing TB incidence. Efforts were needed to improve TB diagnosis. Drug resistance surveillance data were limited, and few trends were available from the WHO/AFR which was particularly concerning given the need for information about multidrug-resistant TB (MDR-TB) in high HIV-prevalence settings.

In August 2005, Ministers of Health from 46 Member States of the WHO/AFR - excluding South Sudan, which was not yet independent at the time - unanimously declared TB an emergency in the Region a response to an epidemic in which the annual number of new TB cases in most African countries had more than quadrupled since 1990 [15]. The declaration urged countries to develop and implement emergency strategies and plans to control the worsening epidemic including laboratory strengthening.

### The Stop TB Strategy (2006–2015)

The Stop TB Partnership formulated a Global Plan to Stop TB for 2006–2015 [16], building on its initial plan for 2001–2005 [17]. This Plan aimed to reduce TB incidence by 2015, aligning with the Millennium Development Goals (MDGs), and to halve TB prevalence and mortality rates compared to 1990 levels. This strategy was supported by a detailed analysis of the necessary strategies, actions, and resources for the next decade [16]. Key activities included expanding geographic coverage of diagnostics to improve case detection, increasing the number of laboratories capable of bacterial culture and drug susceptibility testing, and scaling up new diagnostic tests.

Despite the expansion of laboratory networks, many countries in the WHO/AFR needed improvements in TB laboratory services. Key areas requiring attention included national TB reference laboratories (NTRLs), EQA, and the development of capacity and infrastructure for culture and DST. In some countries, issues such as poor-quality laboratories and inappropriate diagnostic and treatment procedures for MDR-TB patients outside NTPs were prevalent. Implementing new WHO guidelines on the programmatic management of DR- TB was part of the solution [18].

**Fig 1:**
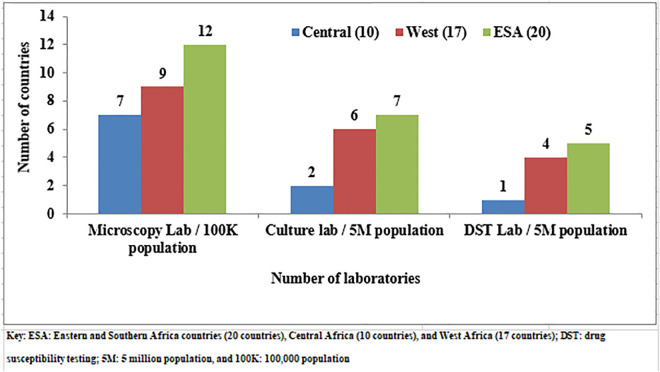
Number of microscopy, culture and DST laboratories per 100K and 5M population by the end of 2015 in the WHO/AFR [19,20]. Key: ESA: Eastern and Southern Africa countries (20 countries), Central Africa (10 countries), and West Africa (17 countries); DST: drug susceptibility testing; 5M: 5 million population, and 100K: 100,000 population.

By the end of 2015, the benchmark set in the Global Plan to Stop TB was reached only for microscopy. In the WHO/AFR, 28/47 (59.5%) Member States achieved the WHO benchmark for microscopy laboratory coverage of one per 100,000 population, while 15/47 (32%) achieved the benchmark of one culture laboratory per five million population, and only 10/47 (21.3%) achieved the standard for one DST laboratory per five million population [19,20] (**Fig 1**).

**Fig 2:**
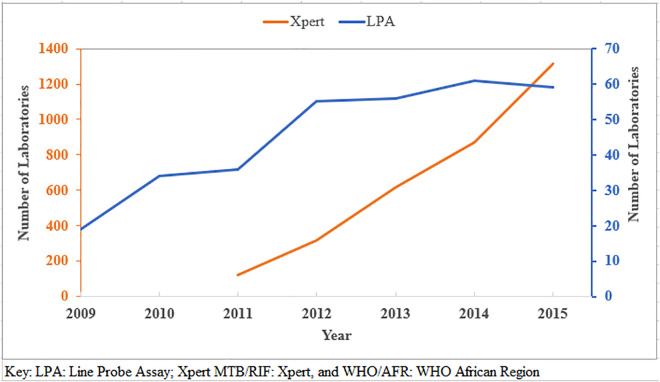
Number of LPA and Xpert MTB/RIF labs in WHO/AFR [19,20]. Key: LPA: Line Probe Assay; Xpert MTB/RIF: Xpert, and WHO/AFR: WHO African Region.

Molecular tests, including Line Probe Assay (LPA), and Xpert MTB/RIF, were endorsed by WHO in 2008 [21], and 2010, respectively [22]. By the end of 2015, around 60 laboratories (in 24 countries), in the WHO/AFR had LPA capabilities, and 1,300 laboratories were equipped with GeneXpert instruments in 40/47 (85.1%) countries [19,20] (**Fig 2**).

The importance of antiretroviral therapy (ART) cannot be overstated. The scale-up of ART has significantly influenced tuberculosis (TB) incidence in Africa, particularly during the Stop TB Strategy era. ART reduces the risk of developing active TB among people living with HIV (PLHIV) by restoring immune function. As ART coverage expanded across the region, TB incidence among PLHIV declined markedly. Countries such as South Africa, Uganda, and Kenya reported measurable reductions in TB cases as ART became more widely available and integrated with TB services [23].

## The present

### The End TB Strategy (2016–2035)

From a laboratory perspective, the WHO’s End TB Strategy [7] prioritizes early diagnosis of TB to achieve universal access to WRDs, increase bacteriologically confirmed TB and drug resistance detection, and reduce the time to diagnosis [24,25].

Achieving these goals requires NTPs to have a quality-assured laboratory network equipped with rapid diagnostics. The Framework of Indicators and Targets for Laboratory Strengthening under the End TB Strategy (2016–2025) [26] was created to evaluate programs’ availability to accurately and quickly detect TB using WRDs, provide universal DST, and maintain testing quality. This framework includes 12 core indicators that WHO will monitor globally as countries work towards meeting the targets [24,25].

Despite progress made in strengthening TB laboratories in the WHO/AFR, significant gaps remain between TB incidence and notification, notified TB cases and those bacteriologically confirmed, and bacteriologically confirmed TB cases and those undergoing first and second-line DST [1].

According to the 2024 WHO Global TB Report, the African Region has recorded the steepest global decline in TB deaths, with a 42% reduction in mortality and a 24% decline in TB incidence between 2015 and 2023 [27]. These achievements are attributed to improved case detection, expanded treatment coverage (from 55% to 74%), and increased national efforts to scale up WHO-recommended interventions. Notably, South Africa surpassed the 2025 milestone by achieving a 50% reduction in TB incidence, while Mozambique, Tanzania, Togo, and Zambia met the target of a 75% reduction in TB deaths [27]. However, catastrophic costs continue to affect 68% of TB-affected households, driven by out-of-pocket expenses, income loss, and limited social protection.

Access to rapid diagnostics has improved from 24% in 2015 to 54% in 2023 but remains insufficient to curb the spread of multidrug-resistant TB (MDR-TB), with over half of MDR-TB cases undiagnosed and untreated. Additionally, the African Region faces a US$3.6 billion annual funding gap, with only US$0.9 billion currently available out of the US$4.5 billion needed for comprehensive TB services.

While the African Region has made commendable progress in reducing TB incidence and mortality, the persistent economic burden, diagnostic gaps, and funding shortfalls pose significant barriers. Urgent action is needed to accelerate efforts, close gaps, and ensure that the region remains on track to meet the End TB Strategy targets by 2025 and beyond.

The COVID-19 pandemic has had a significant and lasting impact on TB control efforts, particularly in the WHO African Region. Disruptions to health services led to declines in TB case detection, treatment initiation, and surveillance. According to the 2024 WHO Global TB Report, TB incidence increased globally in both 2021 and 2022, reversing years of steady decline [27]. In Africa, the pandemic exacerbated existing challenges such as limited diagnostic access and funding gaps. However, countries responded by leveraging existing TB and HIV laboratory networks for COVID-19 testing, strengthening integrated diagnostic platforms, and restoring TB services through catch-up campaigns and digital health tools. These mitigation measures have helped the region recover some lost ground, but accelerated efforts are still needed to meet the 2025 End TB Strategy targets.

## Global and regional-driven initiatives

### The Global Laboratory Initiative (GLI): Global Approach

In 2007, the Global Laboratory Initiative (GLI) was formed as a working group within the Stop TB Partnership [28], with its secretariat located in the WHO Global TB Programme [29]. The GLI is a network of international partners committed to accelerating and expanding access to quality-assured laboratory services to address the diagnostic challenges of TB, particularly HIV-associated and DR-TB. The GLI’s mission is to act as a collaborative platform for the development and dissemination of practical guidance and tools to build and sustain high-quality TB diagnostic networks. While GLI’s support was global, there was a requirement for a regional initiative in Africa to address the continent’s specific needs.

### The Global Laboratory Initiative for Africa (GLI Africa): Regional Approach

In 2013, GLI Africa was established as a regional partnership with its secretariat in the African Society for Laboratory Medicine (ASLM). Its main goal is to coordinate and address challenges related to TB laboratory services in the Region. GLI Africa assists countries through its network of national TB programs and laboratories, multilateral agencies, development and technical partners, public health institutions, and other key stakeholders to improve TB laboratory and diagnostic services in Africa [30]. GLI Africa aligns with several ministerial mandates and recommendations for strengthening laboratory services in the WHO/AFR, such as the Maputo Declaration [31] and the Kigali Declaration on Strengthening Laboratory Management towards Accreditation (SLMTA) [32]. SLMTA programme includes a range of workshops and work-based improvement projects, complemented by site visits and mentoring. The program’s impact is measured using the WHO’s Stepwise Laboratory Quality Improvement Process Towards Accreditation (SLIPTA) [33].]

Following its establishment, one of the GLI Africa secretariat objectives was to coordinate with partners the development of the Regional Framework for Strengthening Tuberculosis Diagnostic Networks in Africa for 2015–2020 [34] which was formally launched in July 2016 through a regional meeting held in Kampala, Uganda [35]. Participants committed to identify priority support areas, leveraging effective partnerships and coordinated technical assistance, and enhance resource mobilization through essential regional interventions to strengthen diagnostic networks. The regional framework has been implemented through two regional TB laboratory projects funded by the Global Fund to Fight AIDS, Tuberculosis and Malaria and led by the WHO SRLs in Uganda and Benin over nearly a decade.

### Birth of the WHO SRL Network: A Global Approach

**Fig 3:**
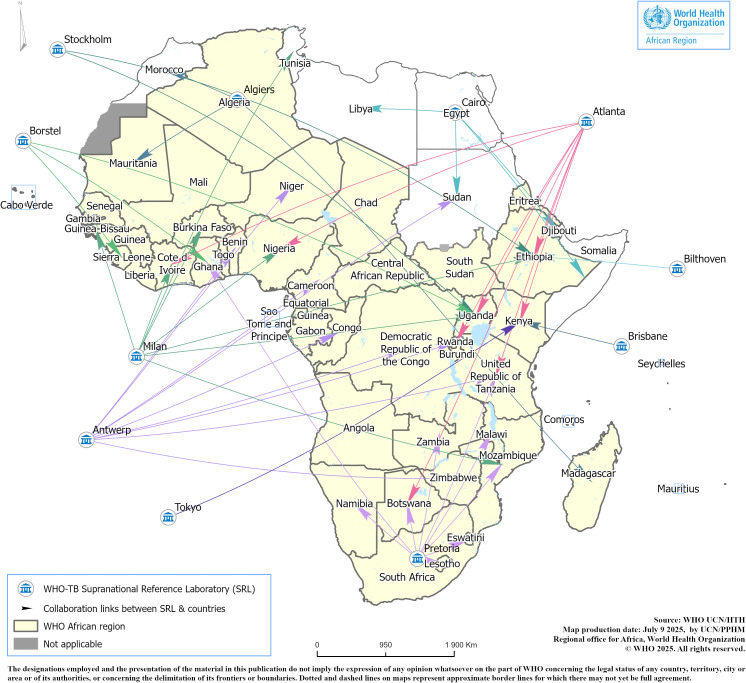
Support of the SRLN to African countries 1994-2012 (unpublished map). Key: SRL Algeria and South Africa were the only SRLs on the African continent supporting a few countries. The others were outside the African region. Source of the basemap shapefile is https://ucn-gis-data-hub-1-1-who.hub.arcgis.com/pages/geographies1.

In 1994, the WHO SRL Network (SRLN) was established to support the WHO-International Union Against TB and Lung Diseases (IUATLD) Global Project on anti-TB drug resistance surveillance, spearhead the introduction of new TB diagnostics, and provide technical leadership to countries in building their laboratory capacity [8,36]. Between 1994 and 2025, under WHO coordination and support, the SRLN expanded from an initial 22 laboratories (mainly outside Africa) that volunteered to form the network based on their institutional capacity and resources to support the Global Project, to 26 SRLs and four National Centers of Excellence (NCE-SRL), driven by regional initiatives and institutional interest in joining the WHO SRLN [37–39]. The support of the SRLN to WHO/AFR countries has been limited to drug resistance projects and quality assurance, as highlighted by Zignol et al. [36] and the impact of that support has been suboptimal in strengthening TB laboratory capacity in the Region. This was mainly due to the lack of a regional-driven laboratory project specifically conducted in the WHO/AFR with a well-coordinated mechanism within the SRLN, rather than the fragmented support that had been provided to a few countries for many years by the SRLs from outside the African continent between 1994 and 2012 (**Fig 3**).

### Expansion of the WHO SRL Network in the WHO/AFR: A Regional Approach

#### Nomination of the SRLs in South Africa, Uganda, and Benin.

##### SRL South Africa:

South Africa’s Supranational Reference Laboratory (SRL), based at the National Institute for Communicable Diseases (NICD), has been a foundational pillar in advancing TB diagnostics across Africa. Originally part of the South African Medical Research Council (SAMRC) Centre for Tuberculosis Research (CTB), the laboratory was among the early contributors to the establishment of the WHO SRL Network in 1994 and was formally designated as an SRL in 2016 [36,40]. SRL South Africa continues to provide technical support to NTRLs in countries like Malawi, Namibia, Botswana, Eswatini, and Lesotho. Its support includes quality assurance, training, and technical support for prevalence, and drug resistance surveys, as well as the implementation of dug susceptibility testing (DST) using new drugs. Leveraging its advanced infrastructure—including biosafety level 3 (BSL-3) facilities and next-generation sequencing—the SRL has led the early adoption of molecular diagnostics such as Xpert MTB/RIF and Xpert MTB/XDR. These tools have accelerated the detection of TB and drug-resistant TB (DR-TB), enabling timely treatment and reducing transmission. Its robust surveillance capacity for resistance patterns to both first- and second-line TB drugs has supported the rapid introduction of novel DR-TB treatments, including bedaquiline and linezolid, by ensuring timely and reliable DST.

Regionally, and more recently, it has played a mentoring role in the development of the Mozambique SRL, helping to expand diagnostic capacity among Lusophone countries.

Through its leadership, innovation, and regional engagement, South Africa’s SRL continues to shape the continent’s response to TB and DR-TB, reinforcing the WHO SRLN and supporting the integration of new SRLs and National Centers of Excellence (NCEs) into the network.

##### SRL Uganda and Benin:

In July 2013 and November 2017, the NTRLs of Uganda and Benin were awarded the status of WHO SRLs and joined the network respectively [41,42]. Both laboratories had been candidates for SRL status for approximately two years for Uganda and five years for Benin, undergoing evaluation by the WHO before achieving full SRL membership. Their modern containment infrastructures, well-trained and committed staff, and strong links with other laboratories in the WHO/AFR countries earned them full SRL status.

### Support of Uganda and Benin SRLs to 45 countries in Africa through the Regional TB Laboratory Projects

The expansion of the SRL network in Africa has facilitated in-country technical assistance to member states, enhancing their laboratory network capacities and contributing to the achievement of the Sustainable Development Goals (SDGs) for universal health coverage.

**Fig 4:**
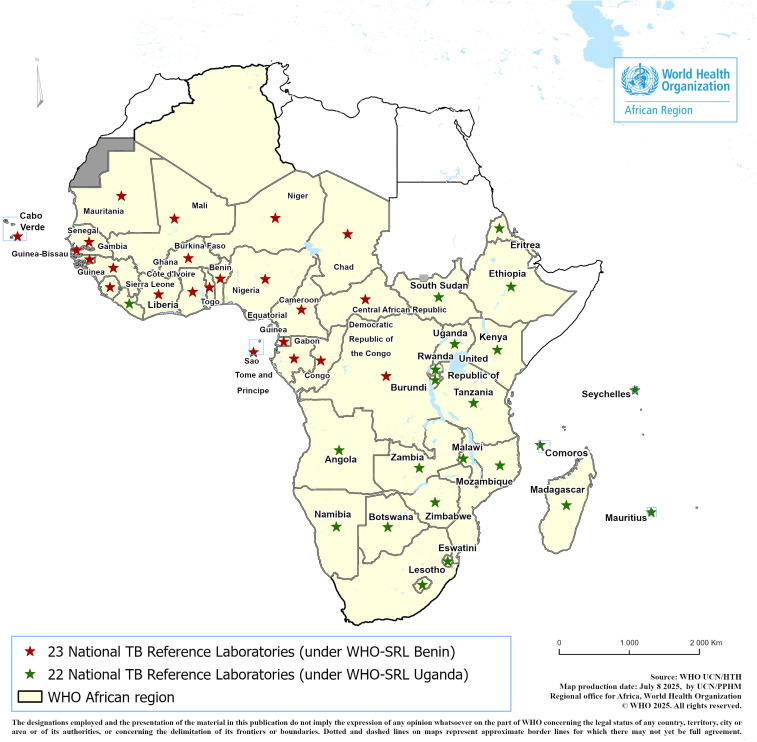
Countries mentored by the SRL Uganda and Benin since 2015 and 2019 respectively (Unpublished map). Key: Only countries enrolled in the TB laboratory projects funded by the GF and coordinated by Benin and Uganda SRLs are represented. Source of the basemap shapefile is https://ucn-gis-data-hub-1-1-who.hub.arcgis.com/pages/geographies1.

In 2015 and 2019, following their nominations, the Uganda and Benin SRLs were respectively awarded Global Fund grants to support a total of 45 countries (22 countries under Uganda, mainly in East and Southern Africa, and 23 under Benin SRL in Western and Central Africa) in the WHO/AFR in improving the capacities of their NTRLs and networks to diagnose TB, Multi/ Extensively Drug-Resistant (M/XDR), and HIV associated-TB (**Fig 4**). In total, two and three phases (three years per phase) of Global Fund grants have been awarded to Benin and Uganda SRLs until December 2024 and June 2025, respectively.

Through Global Fund-supported regional TB laboratory projects, these SRLs have provided technical assistance to 45 countries, strengthening NTRLs, implementing external quality assurance systems, and scaling up WRDs. As a result, the percentage of bacteriologically confirmed TB cases in the WHO/AFR increased from 64% in 2015 to 69% in 2023, reflecting improved access to timely and accurate diagnosis [27].

In parallel, the detection of DR-TB has improved. By 2023, 70% of new and 81% of previously treated TB cases were tested for rifampicin resistance, and 61% of rifampicin-resistant TB cases were tested for fluoroquinolone susceptibility, a critical step in guiding appropriate treatment [27]. These improvements are directly linked to the technical support and capacity-building efforts led by the SRLs in Uganda and Benin, which have helped countries implement molecular diagnostics such as Xpert MTB/RIF and Line Probe Assays (LPAs) and expand culture and drug susceptibility testing services.

Moreover, strengthened laboratory systems have contributed to broader TB outcomes. Early and accurate diagnosis enables timely treatment initiation, reducing TB transmission, lowering mortality, and helping to prevent catastrophic costs for affected households. These outcomes align with the End TB Strategy targets and demonstrate the value of sustained investment in laboratory infrastructure and regional collaboration.

The projects continue to demonstrate their relevance by supporting the differentiated capacity needs of NTRLs in quality assurance (QA), quality management system (QMS) strengthening, implementation of WRDs, DST, Drug Resistance Survey (DRS), Prevalence Survey (PS), TB specimen referral system (TSRS), and laboratory information system (LIS). The projects have also responded innovatively to diagnostic evolutions and emerging needs, including the COVID-19 pandemic, and have been catalytic for many countries in the WHO/AFR [43–45].

### Nomination of Maputo (Mozambique) and Kigali (Rwanda) NTRLs as Candidate SRLs

In March 2021, the NTRLs of Maputo and Kigali were nominated as candidate SRLs by WHO and joined the network. Their nomination was based on their operational capabilities and the requirement to build capacity in performing DST for new anti-TB drugs (e.g., Bedaquiline and Linezolid) and establishing a robust proficiency testing scheme for quality assurance over three years. Both NTRLs were selected to support English and French-speaking countries (Kigali) and Portuguese-speaking countries (Maputo), respectively.

In line with the WHO SRLN expansion plan in the WHO/AFR, large countries with significant populations where the TB laboratory network needs strong support, such as Nigeria and Ethiopia, applied to the WHO to be evaluated for their nomination as National Centers of Excellence (NCE) for their networks. The NTRL of Zaria (Nigeria) and the Ethiopia Public Health Institute (EPHI) in Addis Ababa (Ethiopia) are among those seeking evaluation.

**Fig 5:**
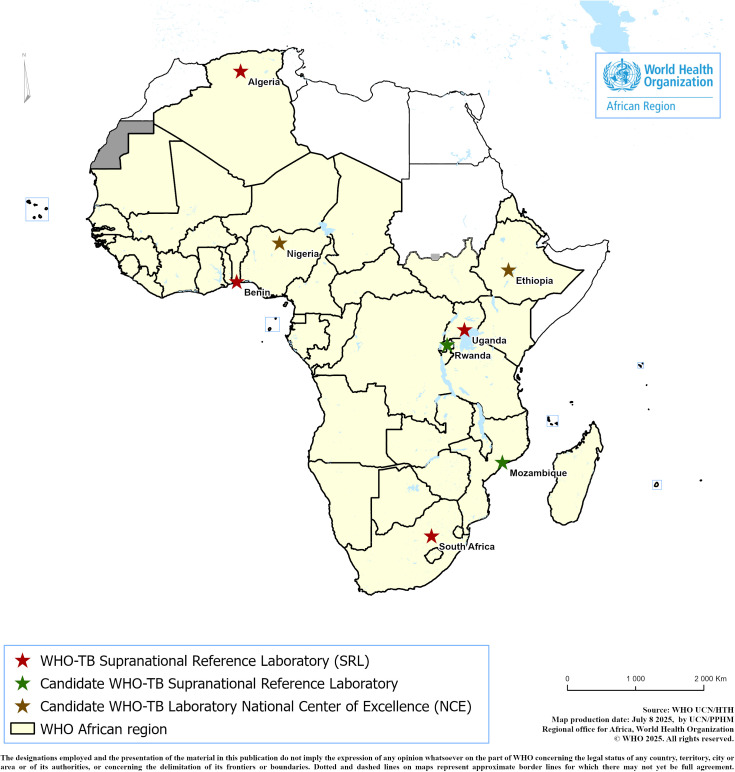
The expansion of the SRL network in the WHO/AFR (unpublished map). Key: NTRLs of Mozambique and Rwanda are candidate SRLs while Ethiopia and Nigeria are candidate National Centers of Excellence. Source of the basemap shapefile is https://ucn-gis-data-hub-1-1-who.hub.arcgis.com/pages/geographies1.

By the end of 2025, WHO plans to evaluate the candidate NTRLs and award them the status of SRL and NCE, respectively. The expansion of the SRLN in Africa (**Fig 5**) will enable countries to establish collaboration agreements with the SRLs of their choice, facilitating timely technical assistance while addressing language and cultural barriers to strengthen south-to-south collaboration and partnership.

## The future perspective

Building on the historical phases of TB laboratory capacity strengthening, the future perspective involves leveraging the established infrastructure and expertise to integrate laboratory systems for diagnosing multiple diseases using the same diagnostic platforms where applicable. This approach aims to enhance efficiency, optimize resource utilization, and improve overall public health outcomes. The DOTS and Stop TB Strategy eras highlighted the importance of establishing robust laboratory networks, improving geographical coverage, and ensuring quality assurance. Despite significant progress, challenges such as limited access to culture and DST services, inadequate funding, and the impact of HIV on TB incidence persisted. The declaration of TB as an emergency by WHO/AFR in 2005 highlighted the necessity for urgent and coordinated action. The End TB Strategy era has seen advancements in rapid diagnostics, universal DST, and quality assurance frameworks. The expansion of the SRL network and the establishment of GLI Africa have provided technical assistance and support to member states, addressing diagnostic challenges and strengthening laboratory capacities [46]. However, gaps remain in TB case detection, bacteriological confirmation, and DST [47].

Investing in TB laboratory networks has proven advantageous in strengthening laboratory systems for other diseases. For instance, the existing TB and HIV laboratory systems facilitated COVID-19 testing across Africa, utilizing shared infrastructure, sample transport systems, and skilled staff [48]. During the COVID-19 pandemic, TB laboratories across Africa were rapidly adapted to support SARS-CoV-2 testing. In Nigeria, for instance, the PEPFAR- and Global Fund–supported TB and HIV laboratory network was leveraged to implement integrated testing for TB, HIV, and COVID-19, using shared molecular platforms and trained personnel [49]. Similarly, the TB laboratory network supported the response to the MPOX outbreak by providing the training and equipment necessary for monitoring the disease. In the case of MPOX, the WHO and Africa CDC mobilized existing laboratory networks, including TB laboratories, to expand diagnostic capacity. Countries like the DRC, Uganda, and South Africa utilized their TB lab infrastructure for Mpox PCR testing, supported by WHO’s interim guidance on diagnostic strategies [50]. During the Ebola epidemic, intensive investment in laboratory testing, contact tracing, and screening was crucial for establishing control over the outbreak [51]. These examples demonstrate that the TB laboratory network’s infrastructure and expertise can be effectively utilized to address various public health challenges. The adaptability of TB laboratory networks in Africa has been demonstrated during major public health emergencies, including the 2014–2016 Ebola outbreak in West Africa, where TB laboratories with biosafety infrastructure were repurposed to support Ebola virus testing, particularly in Guinea, Liberia, and Sierra Leone [52]. These laboratories facilitated rapid diagnosis, contact tracing, and genomic surveillance. For example, in the Democratic Republic of the Congo (DRC), 13 decentralized field laboratories were deployed, enabling the analysis of over 230,000 Ebola samples under stringent biosafety protocols [53]. These examples underscore the value of investing in resilient, multi-disease laboratory systems. The ability to pivot TB laboratory infrastructure and expertise toward emerging threats demonstrates that similar measures—such as decentralization, cross-training of staff, and integration of diagnostic platforms—can be effectively applied to future outbreaks.

The future perspective involves integrating diagnostic platforms and services to simultaneously test for TB, HIV, and other infectious diseases, streamlining laboratory processes, reducing costs, and improving diagnostic accuracy. Continued investment in laboratory infrastructure, training, and quality assurance is essential, along with expanding the SRL network and establishing more laboratories designated as NCEs.

The NCEs in large African countries such as Nigeria and Ethiopia will be nominated based on their strategic importance and capacity to support national TB laboratory networks. While both SRLs and NCEs operate under similar terms of reference, their scope differs significantly. SRLs provide cross-border support and serve as regional hubs for technical leadership, comparative evaluations, and global surveillance activities while NCEs are nationally focused, with their mandate limited to strengthening in-country TB laboratory networks, ensuring national coverage, and supporting internal capacity building in large countries with complex TB epidemiology and vast populations.

Embracing new diagnostic technologies and methodologies, such as molecular tests and digital health solutions, will enhance the speed and accuracy of disease detection. Strengthening south-to-south collaboration and partnerships will facilitate knowledge sharing, capacity building, and resource mobilization. Advocacy for increased funding and supportive policies will be vital to sustain and expand laboratory capacities. Implementing robust monitoring and evaluation frameworks will ensure the effectiveness and efficiency of integrated diagnostic systems. By integrating laboratory systems for multi-disease diagnostics, the future perspective aims to build on past achievements and current advancements, creating a more resilient and responsive public health infrastructure in the WHO/AFR. This holistic approach will contribute to achieving universal health coverage and improving the overall health and well-being of populations.

## Conclusion

The evolution of TB laboratory capacity in the WHO/AFR has shown significant progress, yet challenges remain. Investing in TB laboratory networks has proven beneficial not only for TB control but also for addressing other diseases like COVID-19, MPOX, Ebola among others. This demonstrates the versatility and importance of robust laboratory systems.

Future efforts should focus on strengthening laboratory systems holistically, including infrastructure, training, quality assurance, and cross-cutting laboratory functions. This includes enhancing biosafety measures, implementing robust QMS, and improving specimen referral and transport networks. Expanding the SRL network and establishing more NCEs will provide the necessary support for multi-disease diagnostics and overall laboratory system resilience.

Embracing new diagnostic technologies and methodologies, such as molecular tests and digital/AI-based health solutions, will enhance the speed and accuracy of disease detection. Strengthening collaborations, particularly south-to-south partnerships, will facilitate knowledge sharing, capacity building, and resource mobilization. Advocacy for increased funding and supportive policies will be vital to sustain and expand laboratory capacities.

By building on past achievements and current advancements, integrating laboratory systems for multi-disease diagnostics will create a more resilient and responsive public health infrastructure in the WHO/AFR. This holistic approach will contribute to achieving universal health coverage and improving the overall health and well-being of populations.
